# MrGrid: A Portable Grid Based Molecular Replacement Pipeline

**DOI:** 10.1371/journal.pone.0010049

**Published:** 2010-04-06

**Authors:** Jason W. Schmidberger, Mark A. Bate, Cyril F. Reboul, Steve G. Androulakis, Jennifer M. N. Phan, James C. Whisstock, Wojtek J. Goscinski, David Abramson, Ashley M. Buckle

**Affiliations:** 1 Department of Medical Biochemistry and Biophysics, Karolinska Institutet, Stockholm, Sweden; 2 Department of Biochemistry and Molecular Biology, Monash University, Victoria, Australia; 3 ARC Centre of Excellence in Structural and Functional Microbial Genomics, Monash University, Victoria, Australia; 4 Monash eResearch Centre, Monash University, Victoria, Australia; 5 Clayton School of Information Technology, Monash University, Victoria, Australia; Institute of Molecular and Cell Biology, Singapore

## Abstract

**Background:**

The crystallographic determination of protein structures can be computationally demanding and for difficult cases can benefit from user-friendly interfaces to high-performance computing resources. Molecular replacement (MR) is a popular protein crystallographic technique that exploits the structural similarity between proteins that share some sequence similarity. But the need to trial permutations of search models, space group symmetries and other parameters makes MR time- and labour-intensive. However, MR calculations are embarrassingly parallel and thus ideally suited to distributed computing. In order to address this problem we have developed MrGrid, web-based software that allows multiple MR calculations to be executed across a grid of networked computers, allowing high-throughput MR.

**Methodology/Principal Findings:**

MrGrid is a portable web based application written in Java/JSP and Ruby, and taking advantage of Apple Xgrid technology. Designed to interface with a user defined Xgrid resource the package manages the distribution of multiple MR runs to the available nodes on the Xgrid. We evaluated MrGrid using 10 different protein test cases on a network of 13 computers, and achieved an average speed up factor of 5.69.

**Conclusions:**

MrGrid enables the user to retrieve and manage the results of tens to hundreds of MR calculations quickly and via a single web interface, as well as broadening the range of strategies that can be attempted. This high-throughput approach allows parameter sweeps to be performed in parallel, improving the chances of MR success.

## Introduction

The most common method of protein structure determination is molecular replacement (MR). This involves using the structure of a protein that shares significant sequence similarity with the protein of unknown structure as a starting point in the structure determination. The process involves four steps: (1) Using sequence-comparison methods such as PSI-BLAST [1] to identify suitable structures that can be used for MR; (2) modification of structures (e.g., removal of flexible loop regions and non-identical side chains), to yield search models; (3) Finding the orientation and position of the search model in the unit cell of the target crystal; (4) Refinement of the model using iterative model-building and maximum likelihood atomic refinement. Although there are other methods of structure determination, molecular replacement is predicted to become an increasingly common technique, for two reasons. First, as the number of new folds reported in the Protein Data Bank (PDB) is decreasing, it is increasingly likely that the unknown target structure will belong to a known fold. Second, the emergence of more sophisticated sequence searching algorithms, such as profile-profile matching [2], improve the probability of finding a suitable search model, even in cases of very low similarity (<20% identity). Finally, the MR algorithms are steadily improving.

Where the sequence similarity between the unknown target and the search model is high (sequence identity >40%) the success rate of MR is very good, even without optimisation of the search model. However, in cases of low similarity (identity <30%) MR, and subsequent structure refinement becomes non-trivial, and can require more complicated strategies to effect a solution. Bearing this in mind, there are several criteria that affect the outcome of the MR calculation; 1) structural similarity between search model and target structure (measured by root mean square deviation (RMSD)); 2) percentage of residues missing from the search model (coverage); 3) the amount of conserved side chains (those expected to remain structurally conserved, e.g., in the protein interior). These factors, and thus the outcome of the MR calculation, can be influenced by improvement of the search model. The simplest approach is to remove regions of the structure that are predicted to be different in the search model and target. Within a conserved protein family, the largest structural deviations are typically seen within the loop regions. Therefore, these regions are the first candidates for removal from the search model. However, this process is a subjective one and relies on sequence alignments, which are often incorrect, particularly at low sequence identity. Thus it is often unclear which loops should be removed and, furthermore how much of the loop should be removed. Each edited model must be tested individually in the lengthy structure determination process, with no indication until the later stages of refinement that the structure should be abandoned.

In addition to edits to the search model, other parameters can greatly influence the outcome of a MR calculation. For instance, the presence/absence and handedness of screw axes will remain unknown until a final structural solution is found and several alternatives must be tested in the MR calculation. In addition the estimated RMSD between the search model and unknown structure can affect the outcome of the MR calculation, leading in the worst case to probable solutions being missed. Therefore, the combination of multiple models, space groups and RMSD values makes MR time and labour intensive, and puts an emphasis of the availability and power of computational resources. In order to address this problem we have developed MrGrid, web-based software that allows multiple MR calculations, using the program PHASER [3], to be executed across a grid of networked computers, allowing high-throughput MR.

## Methods

### Mr Grid Overview

MrGrid is a portable web based application written in Java/JSP and Ruby, and taking advantage of Apple Xgrid technology. Designed to interface with a user defined Xgrid resource the package manages the distribution of multiple MR runs to the available nodes on the grid and reports all returned results. Utilizing the maximum likelihood based molecular replacement program PHASER [3], MrGrid enables the user to retrieve and manage the results of tens to hundreds of MR calculations quickly and via a single web interface, as well as broadening the range of strategies that can be attempted, increasing the likelihood of success.

### Using Mr Grid to perform parallel MR on a local network

**Figure 1:**
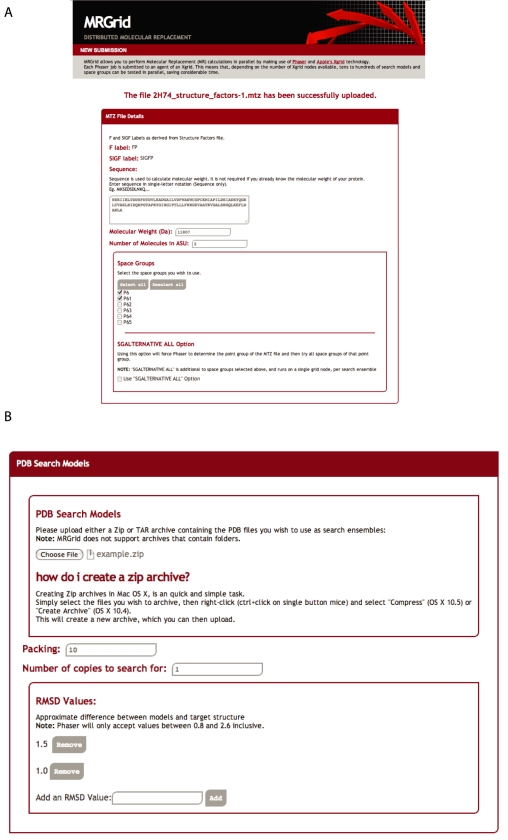
MrGrid web interface showing user input for (a) MTZ file, sequence and space group(s); and (b) search model(s) and RMSD values.

MrGrid is distributed as a self-contained software package, and downloaded and executed across a local (user managed) grid resource. Once setup MrGrid is accessed through a web portal (Figure 1). Apple Xgrid software is preinstalled on Apple operating systems OS X 10.4–10.6, allowing machines to be configured as Xgrid clients by simply ticking a box in system preferences. By default MrGrid processes on the client are given low priority, such that the client remains fully responsive. The remaining requirement is a networked machine running Mac OS Server 10.4–10.6, which acts as the Xgrid controller. The ease of setup of Xgrid is a distinct advantage in setting up MrGrid.

MrGrid will first request that the user uploads the processed structure factor data (in MTZ format) for the MR calculation (Figure 1a). The file is uploaded and parsed to extract the space group (SG) & possible “F” & “SIGF” labels contained within the file. In the case that there is more than one possibility for “F” & “SIGF” labels the user is asked to select the appropriate one. The sequence of the unknown protein can be optionally provided and is used to calculate the molecular weight (Figure 1a). The expected number of molecules in the asymmetric unit (ASU) must also be entered. The user is also presented with the expanded point group of the space group that has been extracted from the MTZ file. This allows the user to expand their search, by selecting any number of SG combinations. Alternatively the user can select the “SGALTERNATIVE ALL” check box, which will search all possible point group SGs on a single node for each search model.

Having defined the experimental data, the user must now input the search models; by uploading a compressed format archive file (either zip or tar) containing all search models in PDB format (Figure 1b). The number of copies of the search model to search for is then entered, along with a packing tolerance and RMSD values to test. Before job submission, the user selects the Xgrid resource to use.

MrGrid will then analyse the user's input, breaking it down into smaller jobs and distributing them to available nodes on the grid resource that the user selected. The number of jobs will be equal to the number of space group options selected (note: SGALTERNATIVE is counted as a space group option), multiplied by the number of search ensembles contained within the compressed archive uploaded, multiplied by the number of RMSD options.

The node that the job runs on is passed a small Ruby script, the MTZ file, one of the search models extracted from the compressed archive, parameters that were entered by the user, as well as the space group derived from the MTZ file. The Ruby script writes a PHASER command script in standard CCP4 [4] format, which contains instructions for PHASER to run the job, and is executed by the node. By default, MrGrid will always run PHASER in MR_AUTO mode.

**Figure 2:**
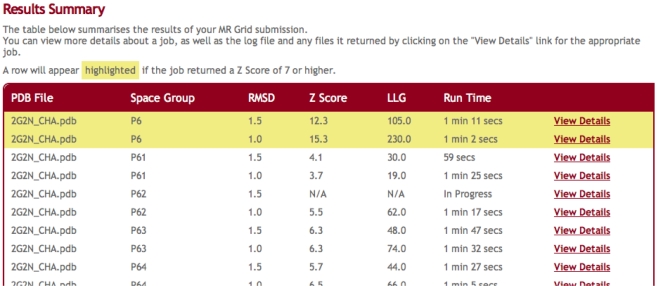
Typical results interface showing PHASER jobs running on Xgrid, allowing the user to view the results of completed jobs independently of others running.

Once submitted to a node, each job runs to completion independent of all other jobs, and a URL where the results of the submission can be accessed is returned to the user. The results page presents a brief summary of the jobs; if a job finds a solution, MrGrid will display the Z-Score & Log Likelihood Gain (LLG) in the job summary (Figure 2). The jobs are also hyperlinked, allowing the user to quickly navigate through the page, the full set of results for a job. The full set of results for a job is made up of several expandable and collapsible elements, from which the user can view/download the output PDB/MTZ files, along with the standard PHASER solution file (.sol), and summary file (.sum), the complete log file for the job can also be viewed. The original MTZ and search model used for the job are also able for download.

### Test Case Selection

A set of 10 proteins were used as test cases, representing 8 different SCOP [5] families (Table 1), and allowing for the parallel execution of 4 to 54 jobs at any one time. PDB entries were selected on the basis of having 3 or more homologous structures in the PDB, with datasets from a range of point group symmetries (Table 1). Both coordinate and structure factor information was retrieved from the PDB for each protein, along with peripheral information necessary for running the MR experiments (e.g., sequence, ASU content). Homologues for each test protein were identified through a BLASTP search of the PDB using the NCBI server (http://www.ncbi.nlm.nih.gov/). MR search models were generally chosen on the basis of a >30% sequence identity across the majority of the monomer of interest (i.e. no partial matches). The exception was the hypothetical protein TTHA0727 test case, which represented cases of lower identity (<30% ID) along with some examples of subdomain insertion within the chosen search models, relative to the test case protein (PDB ID 2CWQ). The purpose of this example was to provide a non-trivial MR example using a divergent group of proteins (from the AhpD-like Superfamily [6]).

**Table 1:** List of test case proteins extracted from the Protein PDB.

|PDB ID	Protein Name	Space Group	Resolution Limit (Å)	Molecular Mass (Da)
2GPZ	Transthyretin-like protein	*P*6	2.5	12700
2NO4	Haloacid Dehalogenase	*P*3_1_21	1.9	24000
2CWQ	Hypothetical protein TTHA0727	*P*3_1_21	1.9	12581
2ENX	Mn-dependant inorganic pyrophosphatase	*H*32	2.8	33597
2RH5	Adenylate kinase	*C*222_1_	2.48	23231
1S3G	Adenylate kinase	*P*3_1_21	2.25	23888
2JCB	5-Formyl-tetrahydrofolate cycloligase	*P*1	1.6	23385
2H74	Thioredoxin	*P*6_1_	2.4	11807
1FB0	Thioredoxin	*P*3_1_21	2.26	11782
2MM1	Myoglobin	*P*3_2_21	2.8	17184

Details about respective datasets are also listed.

## Results and Discussion

### Xgrid-accelerated parallel MR using test cases

**Figure 3:**
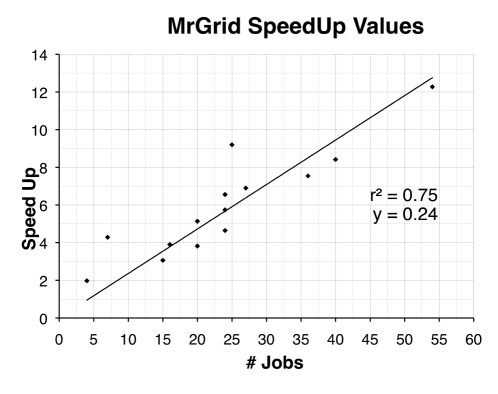
Graph depicting the linear relationship between the numbers of jobs submitted to the Xgrid and the respective speed up values. Speed-up is calculated by dividing linear run time by MrGrid total run time. Linear run time is defined as the sum of the run times of all jobs (job1_runtime + job2_runtime + jobN_runtime). MrGrid total run time is defined as the time difference between the start of the first job and the end of the last job (jobN_finish - job1_start). The linear run time is intended to provide an estimation of how long jobs would take to run synchronously on one computer. *r* ^2^ represents the ‘goodness of fit’ of the linear regression line to the data points. *y* is the intercept on the y axis.

The purpose of this phase of testing was not to assess the capacity of PHASER to perform MR. Rather, it was our intention to simply investigate the advantage to using MrGrid when screening multiple PHASER jobs at one time. Experimental data (structure factors) taken from PDB for the 10 proteins listed in Table 1 were each used in test case experiments on MrGrid in order to demonstrate the utility of the system under standard MR situations. For each protein example, data were screened against each homologue search model (including self), searching all alternative SGs belonging to the reported point group (Table 2). The number of jobs submitted to our local Xgrid (Table 3) varied between 4 and 54, and the corresponding speed up factors showed a clear linear relationship with a correlation of 0.85 (Figure 3). Featuring an average speed up value of 5.69 across all the tests, it is clear that MrGrid has the capacity to significantly reduce the time taken to achieve a MR result when screening numerous parameters.

**Table 2:** Summary of MrGrid results for 10 test cases studied.

PDB ID	# SGs in Point Group	# Search Models	# Jobs	Linear Time (mins)	MrGrid Time (mins)	Speed Up Factor
2JCB	1	4	4	40.63	20.60	1.97
2ENX	1	7	7	17.65	4.12	4.28
2NO4	3	5	15	1339.22	437.92	3.06
2RH5	2	8	16	98.32	25.20	3.90
2CWQ	3	8	24	1424.5	309.50	4.64
2GPZ	6	4	24	76.89	13.40	5.74
1S3G	3	8	24	438.90	66.97	6.55
1FB0	3	9	27	204.57	29.67	6.89
2MM1	3	12	36	80.68	10.7	7.54
2H74	6	9	54	272.47	22.20	12.27

Note – Search model count includes a ‘self’ model, which was the actual protein being investigated.

**Table 3:** Specifications of the Xgrid resource utilized during this study.

Machine #	Machine type	Operating System	Processors (GHz)	RAM (GB)
1	G4 iMac	OS X 10.5.2	1.42	1
2	G5 iMac	OS X 10.4.11	2	2
3	Intel iMac	OS X 10.4.11	(2×) 2.16	2
4	G4 MacMini	OS X 10.5	1.42	1
5	Intel iMac	OS X 10.4.11	(2×) 2.16	2
6	Intel Quad core Duo	OS X 10.4.11	(8×) 3	8
7	Intel MacBook	OS X 10.4.11	(2×) 2	2
8	G5 iMac	OS X 10.4.11	(2×) 2	2
9	G5 iMac	OS X 10.5.2	(2×) 2	2
10	Intel iMac	OS X 10.4.11	(2×) 1.83	2
11	G5 iMac	OS X 10.4.11	1.8	2
12	Intel iMac	OS X 10.4.11	(2×) 2	2
13	Intel iMac	OS X 10.5.1	(2×) 2.4	2
Total			65.54	30

It should be noted that at particular times some workstations were in use by their operator and thus unavailable to Xgrid.

Though a more exhaustive testing may reveal a levelling off of the speed up factors as the number of jobs exceeds the capacity of the grid, the results depicted in Figure 3 display a clear advantage up to 54 jobs when run on our local Xgrid (Table 3). While it is important to note that any particular test case will always run as long as its longest job, in addition to speeding up MR calculations MrGrid provides a convenient solution to screening MR input parameters via a simple web page. It is important to differentiate between making use of spare CPU cycles on desktop computers, as we use here, to form ‘desktop grids’, and dedicated cluster nodes. Performing our experiments on dedicated cluster nodes would clearly increase the efficiency of the calculations.

### Example of an evasive MR solution

We set out to test the utility of the MrGrid approach for a challenging MR case where the sequence similarity of available search models is relatively low (<25%). We chose the peroxidase-related protein yp_604910.1 from *deinococcus geothermalis* (PDB ID 2OYO). A globular all-alpha helical protein, it features two monomers in the ASU. It structure was determined by MAD to 1.52 Å resolution (unpublished). After performing a sequence similarity search using FFAS [2] we identified two potential search models, with sequence identities of 19% (2GMY) and 24% (2O4D). We generated “mixed” models of each (consisting of conserved side chains - all other non alanine/glycine residues truncated at Cγ atom) using the SCRWL server [7], as well as poly-Ala models with and without loop regions. This generated a total of 6 search models, which were input into MrGrid. Further screening against 5 RMSD bins generated 30 separate runs of PHASER, looking for both monomers in the ASU. The majority of calculations took >5 hours to complete. In order to assess whether solutions would refine using standard procedures, we input all solutions having Z scores greater than 7.0 into the refinement program REFMAC [8] and the automatic building and refinement program ARP/wARP [9]. From the 7 solutions tested only one solution (Z score = 9.2) produced a substantial decrease in *R*_free_ (initial = 56%, final = 49%) and successfully built to near completion in ARP/wARP.

The value of the MrGrid parallel approach is that it offers considerable timesavings, such that potential solutions can be tested relatively quickly. In this particular case, performing the MR calculations allowed all 7 potential solutions to be tested in a standard refinement procedure in a matter of hours. In contrast, this would most likely have taken significantly longer (e.g. days-weeks) using a serial approach, with the sole solution perhaps only being identified by chance after a significant period of time.

This paper reports the development of a new web portal MrGrid, which allows multiple PHASER MR calculations to be performed in parallel over networked computers typically available in protein crystallography laboratory. With a demonstrated capacity to significantly reduce the time taken to screen numerous MR jobs, MrGrid is able to facilitate difficult MR cases. Furthermore, parameters sweeps have the capacity to improve the chances of obtaining MR solutions, thus accelerating the structure elucidation process.

### Availability and Future Directions

MrGrid is freely available from http://code.google.com/p/mrgrid/. There are currently efforts to extend MrGrid to non-Apple computing resources, for example using the CONDOR project (http://www.cs.wisc.edu/condor/). In addition, we are also investigating ways of implementing automatic post MR model refinement to provide an automatic method of validation.
